# Pharmacological Strategies for Cataract Management: From Molecular Targets to Clinical Translation

**DOI:** 10.3390/ijms26125658

**Published:** 2025-06-13

**Authors:** Laura de Diego-García, Raquel Rejas-González, Ignacio Cereza Latre, Ana Guzman-Aranguez

**Affiliations:** 1 Department of Biochemistry and Molecular Biology, Facultad de Óptica y Optometría, Universidad Complutense de Madrid, 28037 Madrid, Spain; laura.dediego@vet.ucm.es (L.d.D.-G.); ignaciocereza@gmail.com (I.C.L.); 2 Chronic Disease Programme, UFIEC, Instituto de Salud Carlos III, 28220 Majadahonda, Spain; raquel.rejas@isciii.es

**Keywords:** cataract, pharmacology, oxidative stress, antioxidant, aldose reductase inhibitors, protein aggregation inhibitors, drug delivery

## Abstract

Cataracts, characterized by the opacification of the eye lens, remain a leading cause of reversible blindness globally. Age and diabetes are key risk factors, and with the increasing aging and diabetic population, the global burden of cataracts is projected to rise significantly. Current treatment is predominantly surgical; however, pharmacological strategies could offer a non-invasive alternative with the potential to delay, prevent, or even reverse cataract progression. Recent research has enhanced our understanding of cataractogenesis, emphasizing oxidative stress as a key underlying mechanism, but also including other processes such as calcium dysregulation and altered lens homeostasis or specific events induced by hyperglycemia in diabetic cataracts. New therapeutic approaches have emerged considering the molecular mechanisms involved in cataracts, most of which focus on pharmacological agents with antioxidant properties. Additionally, small-molecule chaperones, aldose reductase inhibitors, and protein aggregation inhibitors have also demonstrated potential in stabilizing or restoring lens protein structure and transparency. While experimental results have shown encouraging results, further research is needed to optimize drug delivery systems to the lens, assess long-term safety, and confirm the clinical efficacy of these treatments. This article reviews current progress in pharmacological treatments for cataracts, outlining challenges and prospects for future integration into clinical practice.

## 1. Introduction

The crystalline lens serves as an important refractive element and ultraviolet (UV) filter. The loss of lens transparency leads to the development of cataracts. The opacity of the lens can result from metabolic, nutritional, and environmental changes. Cataracts are the leading cause of reversible blindness worldwide. In 2020, an estimated 79 million people over the age of 50 were classified as having moderate to severe visual impairment, with approximately 15 million individuals classified as blind due to cataracts globally [1]. With the increasing global aging population, the number of people blind from cataracts is projected to rise to 50 million by 2050.

Currently, the primary approach for cataract treatment is surgery and the implantation of an intraocular lens. As with any surgical procedure, cataract surgery carries a risk of complications. Intraoperative complications may include capsular rupture, loss of nuclear material into the vitreous cavity, and vitreous loss. Postoperative complications may also exist, including endophthalmitis, posterior capsular opacification, refractive errors, or dry eye. Even though, nowadays these complications are infrequent, they can alter visual outcomes and life quality. On the other hand, given the increasing aging of the population, a continuous increase in cataract patients is expected in the coming years, leading to a significant economic cost for public health systems. Thus, the demand for cataract surgery continues to exceed the capacity of limited public health resources, particularly in developing countries. Notably, it has been suggested that delaying the onset of cataracts by just 10 years could reduce their incidence by half [2]. Moreover, achieving satisfactory visual outcomes is challenging in pediatric cataracts even with early surgery [3]. In this context, the development of new pharmacological therapies for the prevention and/or treatment of cataracts is essential to lessen the socio-economic burden and enhance patients’ quality of life.

One of the main challenges in pharmacological treatment for cataracts is achieving an effective concentration of the drug in the lens. The lens is a structure without a blood supply that restricts efficient oral or intravenous drug administration. Regarding topical ocular administration, the cornea, located in front of the lens, is a barrier that limits the access of drugs in eye drops from reaching ocular structures. Importantly, as the lens itself is not a uniform tissue and is composed of metabolically and structurally distinct regions, therapeutic strategies must not only ensure lens penetration but also facilitate drug distribution to specific compartments based on cataract type. Addressing these regional differences is crucial for the development of effective pharmacological approaches to cataract prevention and treatment. Additionally, some pharmacological compounds depict low water solubility and generally poor ocular bioavailability, with absorption variability among patients. To address this issue, new nanotechnology-based delivery strategies are being developed.

This paper provides an overview of potential anti-cataract drugs describing their molecular mechanisms of action, discussing their limitations and challenges for clinical translation, and including new drug delivery strategies developed for some of them to improve their bioavailability and efficacy. To better comprehend the potential pharmacological treatments summarized, lens characteristics, the types of cataracts, and the main pathological molecular mechanisms related to cataract formation are first described.

## 2. Lens Characteristics and Cataract Types

### 2.1. Lens Development and Structure

The lens is a transparent, refractive, and avascular structure located posterior to the iris. It plays a vital role in the eye’s optical system by dynamically adjusting its shape to focus light accurately onto the retina.

**Figure 1:**
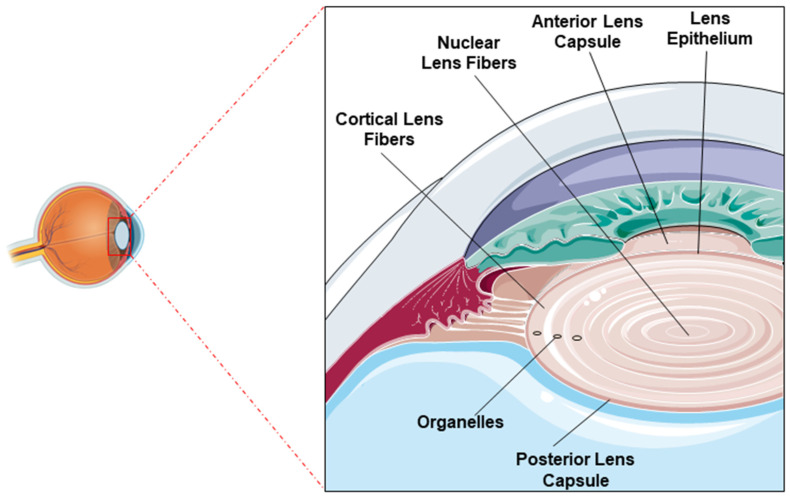
Lens structure: Image showing the normal position of the lens within the eye (**left**) and architecture of the lens indicating the anterior and posterior lens capsules, the epithelial layer, and the nuclear and cortical fibers (**right**).

The lens is composed of three main components: the lens capsule, lens epithelium, and lens fibers (Figure 1). Lens epithelial cells play a crucial role in maintaining the metabolic activity of the lens. They act as a protective barrier against external insults and retain the ability to differentiate into lens fiber cells throughout life. At the lens equator, these epithelial cells undergo differentiation into elongated fiber cells, which are progressively compacted toward the center of the lens (Figure 1). The lens is predominantly composed of fiber cells, with the innermost fibers lacking organelles to maintain transparency. These fiber cells express high concentrations of soluble lens proteins known as crystallins, whose short-range ordered arrangement is indispensable for maintaining the transparency and refractive function of the lens. There are three types of crystallins (α, β, and γ) in the lens, α-crystallins are present in lens epithelial cells and fiber cells whereas β- and γ-crystallins are exclusively expressed in fiber cells. Additionally, α-crystallins act as molecular chaperones, stabilizing partially denatured proteins and preserving their solubility [4].

This structural organization is the result of a highly conserved developmental process. The lens originates from the surface ectoderm during embryogenesis. Posterior epithelial cells elongate to form the primary lens fiber cells, which fill the embryonic lens vesicle. Throughout life, anterior epithelial cells near the equator continuously differentiate into secondary fiber cells, which are added concentrically around the older, centrally located fibers [5]. This lifelong addition creates a characteristic age gradient: the nucleus contains the oldest fibers, while the cortex holds progressively younger ones. These regions are critical for maintaining homeostasis and antioxidant defense, with most of the metabolic activity restricted to the anterior epithelial layer and outer cortical fibers while the nuclear region is more vulnerable to age-related degeneration.

The highly organized cellular architecture, the elimination of organelles during the maturation of fiber cells, and the dense accumulation of crystallins guarantee lens transparency. While these structural adaptations are essential for establishing lens transparency, the lack of a direct blood supply requires a specialized system to preserve cellular integrity and crystalline solubility. To address this, the lens employs a unique microcirculation system sustained by Na^+^/K^+^-ATPase pumps, aquaporins, and gap junctions that generate circulating ionic currents [6]. These currents regulate fiber cell volume, mediate the influx of nutrients, antioxidants, and metabolic substrates into the deeper lens regions, and facilitate the efflux of waste products [6,7]. Any failure in this microcirculatory network—especially in aging—may compromise delivery to the nucleus and contribute to cataractogenesis. Therefore, upregulating this microcirculation pathway may enhance the distribution of endogenous antioxidants and therapeutic molecules to the lens core, representing a novel pharmacological approach to delay the onset and progression of age-related nuclear cataracts.

These developmental, structural, and physiological features may explain the emergence of distinct cataract phenotypes and highlight the importance of tailoring pharmacological strategies to specific regions of the lens.

### 2.2. Cataract Types

**Figure 2:**
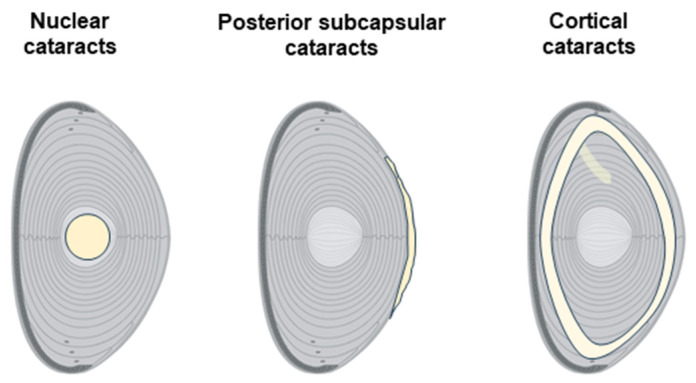
Cataract types according to location in the lens. Schematic diagrams representing the main types of cataracts: nuclear, posterior subcapsular, and cortical.

Lens opacification can occur at different lens regions and is currently used to classify cataracts as nuclear, cortical, and posterior subcapsular (Figure 2).

Nuclear cataracts primarily affect the nucleus or central region of the lens and are typically associated with aging processes. Patients with nuclear cataracts may initially experience improved near vision (a phenomenon known as “second sight”) due to the gradual stiffening of the lens, but eventually, vision becomes blurred due to the continued opacification. Epidemiological studies have associated living in tropical and subtropical regions with an increased risk of developing nuclear cataracts [8].

Cortical cataracts are characterized by the development of wedge-shaped opacities in the outer cortex of the lens. Cortical cataracts often have a slower progression compared to nuclear cataracts, and they are frequently associated with conditions like diabetes mellitus [9]. In the early stages, cortical cataracts may not significantly affect vision, but they can lead to marked visual impairment as opacities encroach upon the visual axis.

Posterior subcapsular cataracts form beneath the lens capsule, at the posterior part of the lens, just in front of its vitreous face. They typically start as a small, opaque spot that grows over time. Posterior subcapsular cataracts are often associated with more rapid progression compared to nuclear or cortical cataracts, and they tend to cause significant symptoms at earlier stages due to their central location within the visual axis, which interferes with both distance and near vision. Posterior subcapsular cataracts are more common in younger individuals, and their development is frequently associated with the use of systemic medications such as diabetes drugs or prolonged steroid use [10].

Furthermore, it is noteworthy that lens opacities may occur simultaneously in multiple regions, potentially complicating the classification and diagnosis of cataract subtypes due to overlapping features.

## 3. Molecular Mechanisms Involved in Cataract Development

### 3.1. Oxidative Stress

**Figure 3:**
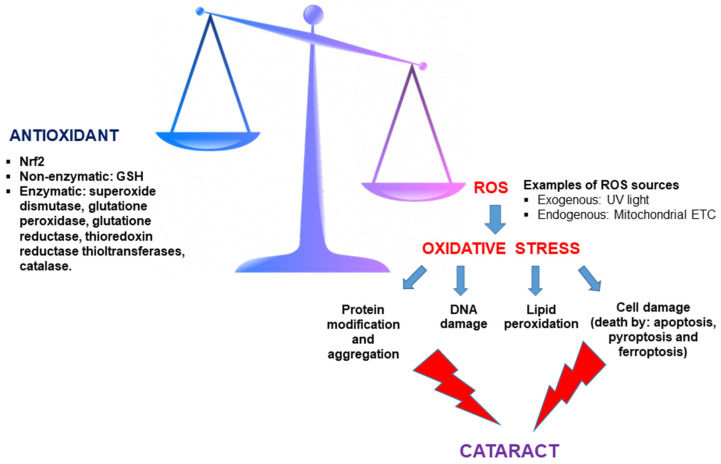
Imbalance between antioxidant defenses and reactive oxygen species (ROS) leads to oxidative stress and cataract formation. Antioxidants, including both enzymatic and non-enzymatic (glutathione, GSH) components, counteract oxidative stress mediated by ROS. Excess ROS—originating from exogenous sources like UV light and endogenous sources such as the mitochondrial electron transport chain—disrupts this balance, triggering oxidative stress. This cascade results in protein modification/aggregation, DNA damage, lipid peroxidation, and various forms of cell death (apoptosis, pyroptosis, and ferroptosis), culminating in cataract formation.

Oxidative stress is a noteworthy factor in the beginning and development of cataracts. This stress arises from the imbalance that can occur between cellular antioxidant mechanisms and the production of so-called free radicals (Figure 3). Free radicals are chemical species (atoms, ions, or molecules) with an unpaired electron in their outermost orbital, giving them an unstable spatial configuration and, therefore, a high reactivity with other substances. In organisms that use oxygen, a category of free radicals called reactive oxygen species (ROS) is generated. Different types of ROS include hydrogen peroxide, hydroxyl radical, or superoxide anion.

In the lens, due to its high metabolic activity, there is a high endogenous production of ROS at the mitochondrial level compared to other body locations [11]. Additionally, UV and visible light are exogenous sources of ROS for the lens. In the lens, the wavelength between 300 and 400 nanometers (nm) is predominantly absorbed by Trp (tryptophan) derivatives: quinurenine, 3-hydroxyquinurenine, and 3-hydroxyquinurenine-O-beta-D-glucoside. These mentioned components are UV light-filtering compounds [12]. Over time, these UV-filtering compounds derived from tryptophan can turn into photosensitizers, generating oxidative damage [12].

With aging, the potential accumulation of ROS in the lens increases, and the concentration of anti-oxidant agents and enzymes decreases, leading to lens damage due to oxidative stress. For example, there is a reduction in the content of reduced glutathione (GSH) [13], the principal non-enzymatic endogenous antioxidant in the lens that protects against chemical and oxidative stress, participating in the prevention of crystalline aggregate formation and the loss of transparency. High levels of GSH (millimolar range) are found in the lens and its biosynthesis is localized to the metabolically active regions of the lens, namely the epithelium and cortex. This results in a concentration gradient of GSH, with the highest levels found in the epithelial and cortical fiber cells, and the lowest levels in the nucleus [14]. This spatial distribution facilitates the passive diffusion of GSH to the nucleus through gap junction channels.

The decrease in GSH with aging particularly affects the center of the lens, with GSH depletion considered a major factor in age-related nuclear cataracts. The age-associated decline in the activity of enzymes responsible for GSH biosynthesis [15] and recycling [16] as well as deficiency in the GSH transport from the cortex to the nucleus by altered gap junctional intercellular communication might be critical factors in the regional GSH depletion related to aging [17]. An alternative and complementary hypothesis proposes that the depletion of GSH in the nucleus may also result from age-related impairments in the lens microcirculation system, which fails to deliver sufficient GSH and other antioxidants to the inner nucleus [14,18].

The antioxidant lens system is also supported by antioxidant enzymes, and it is under the control of transcription factor Nrf2 (nuclear factor erythroid 2-related factor). In the absence of oxidative stress, the inhibitor Keap1 blocks the activation of the Nrf2 protein and causes its permanent degradation. In contrast, under oxidative stress conditions, the oxidation of Keap1 cysteine residues alters its binding to Nrf2, and Nrf2 translocates to the cell nucleus to induce the transcription of antioxidant genes [19]. Nrf2 knockout mice exhibited an amplified incidence and severity of cataracts [20], reinforcing the pivotal role of Nrf2 in oxidative damage and cataractogenesis prevention. Decreased levels of Nrf2 and increased levels of Keap1 together with raised levels of DNA demethylation in the Keap1 promoter have been found in human and bovine aging lenses [21]. Likewise, during the normal process of lens aging, the levels and activity of Nrf2 downstream enzymes, including glutathione reductase, superoxide dismutase, glutathione peroxidase, thioredoxin reductase, and thioltransferases are reduced in lenses [22,23,24].

Thus, aging increases the lens’ susceptibility to oxidative stress, promoting cataract development. Specifically, among the effects triggered by increased ROS in the lens related to cataract development are the oxidation and induction of crystalline aggregation, leading to lens opacity and DNA damage resulting in mutations. It has been reported that in age-related nuclear cataracts, the vast majority of cysteine residues and approximately 50–60% of methionine residues of lens proteins are oxidized [25]. This leads to an increased formation of disulfide bonds and other cross-linkages between proteins, resulting in protein aggregation and light scattering.

ROS can also induce DNA damage and lipid peroxidation. DNA damage can manifest in various forms, including specific modifications such as the oxidation of purine and pyrimidine bases, as well as more extensive structural alterations to the DNA molecule such as strand breaks or sister chromatid exchange. Significantly higher levels of 8-hydroxy-2-deoxyguanosine, which is the most profuse oxidation product in the purine base of DNA and is considered a marker of oxidative DNA damage, were detected in lenses with age-related cataracts compared to control subjects [26]. Lipid peroxidation is an event driven by the free radical oxidation of polyunsaturated fatty acids present in cell membranes, resulting in the generation of peroxyl radicals. This process, when occurring within the lipid bilayer of cell membranes, disrupts lipid-based connections within lenticular fiber membranes. Elevated levels of different lipid peroxidation end products were assessed in the aqueous humor and lenses of diabetic and senile cataract patients [27,28]. Moreover, the extremely reactive peroxyl radicals stimulate autocatalytic chain reactions, resulting in the formation of hydroperoxides, which serve as ferroptosis hallmarks. It has been recently demonstrated that aged lens epithelial cells exhibit a high vulnerability to ferroptosis, suggesting that this cell death mechanism might occur in the human lens during aging and cataractogenesis [29].

Additionally, ROS can also trigger other cell death events in lens epithelial cells, such as apoptosis characterized by upregulation of the pro-apoptotic Bcl-2-associated X protein (*BAX*) gene and downregulation of the anti-apoptotic B-cell lymphoma-2 (*BCL-2*) gene [30] as well as pyroptosis mediated by ROS-induced activation of the NLRP3 inflammasome/caspase 1/gasdermin D pathway [31].

### 3.2. Diabetic Cataract

Diabetes mellitus is one of the major causes of morbidity and mortality worldwide. It negatively affects the body and is characterized by chronic hyperglycemia, which leads to augmented oxidative stress and alterations in the metabolism of various substances such as fats, proteins, and carbohydrates.

**Figure 4:**
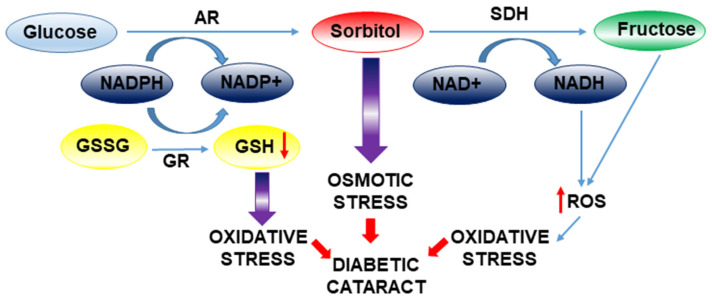
Mechanistic overview of osmotic and oxidative stress in diabetic cataract formation. Osmotic stress is initiated by the activation of aldose reductase (AR), which reduces glucose to sorbitol and is subsequently metabolized by sorbitol dehydrogenase (SDH). Accumulation of sorbitol contributes to cellular osmotic imbalance. Concomitantly, oxidative stress is driven by the excessive generation of reactive oxygen species (ROS) and impaired glutathione reductase (GR) activity, leading to lens damage and cataract development.

It is important to note that cataracts develop more precociously in diabetic patients. The increased activation of the polyol pathway and the accumulation of sorbitol are related to this earlier onset of cataracts in diabetics (Figure 4). Aldose reductase (AR) is a vital enzyme in the polyol pathway and is a significant factor in the pathogenesis of various diabetic complications. Through this pathway, high levels of glucose are reduced to sorbitol in the lens. Subsequently, the enzyme sorbitol dehydrogenase (SDH) must convert sorbitol to fructose, a reaction that also generates NADH (nicotinamide adenine dinucleotide). In diabetic patients, there is an imbalance between sorbitol production and its transformation into fructose, leading to sorbitol accumulation. The accumulation of sorbitol causes osmotic changes that lead to the appearance of swollen (hydrophilic) fibers in the lens, which can break, generating opacities in the lens [32].

Cortical cell swelling in human diabetic cataracts cannot be exclusively ascribed to osmotic stress induced by sorbitol accumulation. The contributions of oxidative stress and protein glycation, which arise from enhanced polyol metabolism and increased fructose production, are likely to play a significant role in the pathogenesis of diabetic cataract formation. Thus, aldose reductase activity consumes NADPH (Figure 4), a critical cofactor necessary for the regeneration of the primary lens antioxidant, glutathione (GSH), from its oxidized form (GSSG). Moreover, the polyol pathway generates an excess of ROS from the transformation of NADH by NAD(P)H oxidase. Additionally, the downstream metabolism of fructose contributes to ROS generation through its consumption of ATP for its conversion to fructose-1-phosphate and fructose-1,6-bisphosphate. The resultant decrease in ATP levels stimulates mitochondrial respiration [33], a process that further enhances superoxide production.

Advanced glycation end product (AGE) production by fructose and its metabolites (3-deoxyglucosone, glyceraldehyde-3-phosphate, and methylglyoxal) is also augmented [34,35]. The formation of AGEs elevates the likelihood of aggregations or cross-linkages between proteins, affecting crystallins [36], and it can further amplify oxidative stress by impairing the function of antioxidant enzymes [37].

### 3.3. Dysregulation of Calcium Content

**Figure 5:**
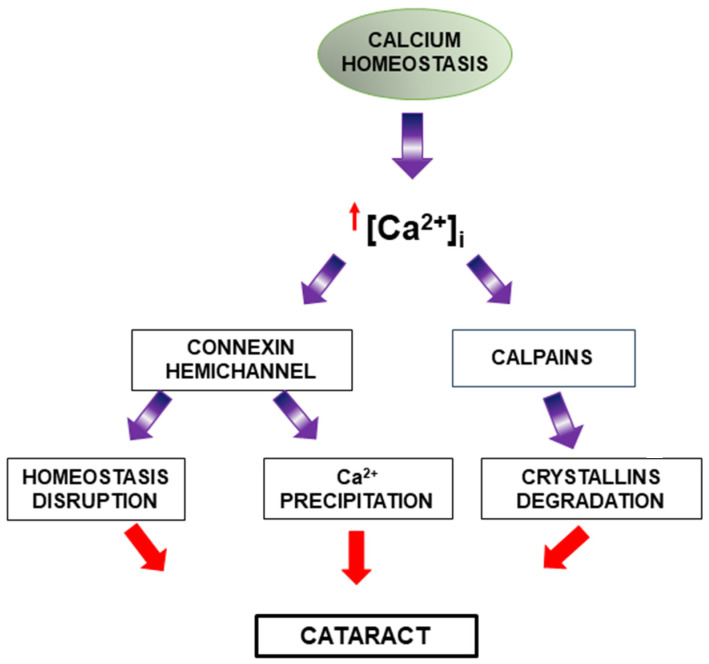
Disruption of calcium homeostasis and its role in cataract formation. Under physiological conditions, calcium homeostasis is tightly regulated. However, elevated intracellular calcium levels lead to pathological outcomes. Increased calcium activates connexin hemichannels and calpains. Connexin hemichannel activation contributes to homeostasis disruption, while calpain activation leads to crystallin protein degradation. These processes collectively disrupt lens transparency and contribute to cataractogenesis.

Maintaining stable calcium levels through calcium pump activity and channels appears to be important to prevent cataract formation. Calcium homeostasis disruption has been associated with lens pathology (Figure 5) and is related to the formation of various types of cataracts, both human and experimental [38]. In this regard, elevated intracellular calcium levels have been detected in cataractous lenses, and more recently, alterations in Ca^2+^ signaling have been determined in human lens epithelial cells during cataract progression [39].

Intracellular calcium overload can generate processes such as the activation of calcium-dependent enzymes like calpains [38]. Calpains are calcium-dependent cysteine proteases that can induce the degradation of crystallins and promote cataract formation [40].

Although high extracellular calcium is known to inhibit hemichannel opening [41,42], several studies have reported that an increase in intracellular calcium can positively regulate connexin hemichannel activity [43,44]. Thus, it has been determined that increased intracellular calcium and calmodulin activation in the lens can activate connexin hemichannels (essential for mediating communication and the exchange of the molecules between cells, as well as between the intracellular and extracellular environments) in lens fibers, disrupting their proper homeostasis and consequently favoring cataract development [45]. In the same way, mutant lens connexins with increased hemichannel activity induced significant increases in intracellular calcium and subsequent cataracts through Ca^2+^ precipitation and biomineralization [46].

## 4. Potential Pharmacological Approaches

### 4.1. Antioxidants

Given the importance of oxidative stress in the pathological mechanisms involved in cataract development, the use of numerous antioxidant molecules for cataract treatment has been considered in recent years (Table 1), although a reduced number of clinical trials have been conducted (Table 2).

#### 4.1.1. Resveratrol

Resveratrol is a polyphenol with antioxidant, anti-angiogenic, and anti-inflammatory properties. Its antioxidant power for cataract treatment has been established in in vitro and in vivo experiments. In primary and immortalized human lens epithelial cells, resveratrol treatment protects against oxidative damage by increasing antioxidative enzyme expression [47] and reducing cellular senescence markers [48]. This last effect seems to be mediated by the activation of forkhead transcription members (FoxO1A, FoxO3A, and FoxO4A). FoxO3A and sirtuin type 1 form a complex within cells against oxidative stress and it is known that sirtuin type 1 activation is a key component in resveratrol’s antioxidant action [49]. Induction of sirtuin type 1 by resveratrol decreased acetyl-p53 levels and abolished apoptosis in human lens epithelial cells challenged with H_2_O_2_ [50]. Interestingly, resveratrol can also promote autophagy by activating the p38 mitogen-activated protein kinase pathway in human lens epithelial cells [51]. The activation of the autophagy process in response to oxidative stress contributes to maintaining intracellular homeostasis in the lens [147]. Additionally, resveratrol inhibited TGF (transforming growth factor)-β2-induced expression of α-SMA (smooth muscle actin), a myofibroblast marker, in human lens epithelial cells and in human capsular bags following simulated cataract surgery. These results suggest that resveratrol could be potentially useful in preventing the epithelial–mesenchymal transition linked to posterior capsule opacification [52]

Regarding in vivo findings, in an experimental cataract model induced by sodium selenite, resveratrol treatment restoredGSH levels and diminished malondialdehyde levels [53]. Moreover, resveratrol at a dosage of 40 mg/kg was also found to moderate cataract occurrence when compared to the group only treated with selenite [53]. A similar dose of resveratrol was effective in attenuating lens opacification in the naphthalene-induced cataract experimental model by preserving antioxidants, Ca^2+^ ATPase function, and protein content and reducing lipid peroxidation [54]. In hyperglycemic streptozotocin-induced diabetic rats, resveratrol ameliorated lens epithelial cell apoptosis [55], and a significant delay of cataract progression was achieved using 30 mg/kg/d of resveratrol [56].

Despite its undoubted interest as an anti-cataract drug, this polyphenol is characterized by rapid metabolism and poor bioavailability [148]. In order to improve its potential therapeutic effect, nanoparticle-based drug delivery systems have been designed. Gold nanoparticles containing resveratrol showed a higher antioxidant capacity and induced a more significant cataract development delay in sodium selenite-induced cataract rats than free resveratrol [57]. Similarly, improved antioxidant activity was detected for resveratrol loaded in lipid–cyclodextrin-based nanoparticles compared to free resveratrol [58]. On the other hand, chitosan nanoparticles loaded with resveratrol worked as inhibitors of γ-crystallin aggregation in in vitro assays [59].

#### 4.1.2. N-Acetylcysteine and Its Derivatives

N-acetylcysteine is an acetylated form of cysteine capable of stimulating glutathione synthesis and increasing antioxidant enzyme glutathione S-transferase activity [60]. In a rat model of selenite-induced cataracts, intraperitoneal injection of N-acetylcysteine has been shown to provide antioxidant-effective protection against cataracts, increasing glutathione levels and reducing malondialdehyde concentration [61]. Only approximately 14% of the animals developed a dense cataract compared to the 50% detected in the untreated rat group [61]. Apart from neutralizing free radicals, N-acetylcysteine also regulates the Foxo3a/TRIM69/p53 pathway attenuating Foxo3a phosphorylation and TRIM69 expression with a subsequent reduction in p53-induced apoptosis [62].

Due to its negative charge, N-acetylcysteine entrance in the lens epithelial cells is challenged. To overcome this limitation, the use of its derivatives, N-acetylcysteine amide (NACA) and (2 R, 2 R′)-3,3′-disulfanediyl bis(2-acetamidopropanamide) (diNACA), has been proposed. NACA exhibited strong antioxidant properties in an animal model of selenite-induced cataracts, increasing GSH concentration, decreasing malondialdehyde levels, and maintaining normal levels of calcium and antioxidant enzyme activities with a significant reduction in cataract severity [63]. Similarly, pretreatment with the compounds NACA and diNACA (both at 10 mM) prevented cataract formation induced by H_2_O_2_ and glucose oxidase exposure in ex vivo porcine lens models and rat lenses [64]. The NACA-induced reduction in lens opacity observed was associated with elevated levels of cysteine and GSH, indicating an antioxidant mechanism involving thiol enhancement. Surprisingly, although diNACA treatment reduced lens opacities, it did not significantly increase the levels of cysteine, cystine, or GSH, suggesting that it may exert its effects through a distinct antioxidant pathway that remains elusive [64].

#### 4.1.3. Pirenoxine

Pirenoxine is a xanthomatin that possesses strong antioxidant properties and can slow down lens opacity as revealed in several in vitro and in vivo studies [65,66,67,68]. Pirenoxine increased GSH levels and superoxide dismutase and catalase activity in the lens and the serum in experimentally induced cataract rats [68]. This agent also reduced lipid peroxidation product concentration [65]. Additionally, in an animal model of selenite-induced cataracts, pirenoxine inhibited the combination of quinoid substances with the sulfhydryl groups of lens proteins and the subsequent protein aggregation, delaying the loss of lens transparency [67]. Pirenoxine was marketed in Japan with the brand names Catalin© and Kary Uni©, available as eye drops. However, it is primarily used off-label for age-related cataracts and its clinical efficacy remains a subject of debate due to inconsistent and controversial findings from clinical studies. Clinical trials have reported the effectiveness of Catalin eye drops in inhibiting opacity and progression of cortical cataracts [69,70]. Densitometric analysis of lens transparency indicated that patients treated with pirenoxine showed a minor optical density in the anterior and posterior cortical lens layers and underneath the posterior capsule in the first months of treatment compared to cataract patients and the transparency remained unchanged in the other lens layers [70]. The patients did not experience any adverse effects with the treatment, and the drug’s effectiveness in delaying cataract progression was greater in younger patients, up to 59 years old [69]. On the contrary, other clinical assays concluded that this drug was not able to decelerate cataract progression [71]. In this last study, patients with different patterns of lens opacity were included, whereas in the studies showing positive effects of pirenoxine, mainly cortical cataracts were evaluated. These results suggest cataract-type-specific effects for pirenoxine and highlight the need for additional clinical studies to corroborate the usefulness of pirenoxine in cataract treatment.

#### 4.1.4. L-Carnosine and N-Acetylcarnosine

L-carnosine is a dipeptide containing amino acids (β-alanyl-L-histidine). Its potential as an anti-cataract agent is due to its antioxidant properties, glycation inhibition [72], and blocking of calpain-mediated proteolysis [73]. Indeed, its efficacy in cataract prevention has been demonstrated in several models of cataracts including radiation-induced cataracts, and age-related and diabetic cataracts [73,74,75]. Despite its anti-cataract effect, L-carnosine exhibited a scarce ability to penetrate the cornea, precluding its therapeutic use in ophthalmic diseases. A lipid-based system containing hyaluronic acid has been employed to circumvent this drawback [79]. This complex showed controlled corneal permeation of L-carnosine with good biosafety and significant inhibition of lens browning and advanced glycation end products [79].

Alternatively, the use of the prodrug N-acetylcarnosine has been considered since it can pass more easily through the cornea and be metabolized into L-carnosine. In addition, to increase corneal passing of N-acetylcarnosine, this prodrug has been also loaded in gold nanoparticles with good results in terms of biosafety and bioavailability [80].

Metabolic generation of L-carnosine from N-acetylcarnosine requires adequate bioactivity of the lens enzyme system. Lens enzyme activity and normal lens function can be altered in impaired cataract lenses [149], which might affect N-acetylcarnosine metabolism and the therapeutic efficiency of this prodrug. Despite this potential obstacle, topical application of N-acetylcarnosine was examined in clinical trials reporting worthy data. Cataract patients treated with 1% N-acetylcarnosine over 6 and 9 months showed significantly lower lens opacity, reduced glare, and an improvement in visual functions compared to baseline [76,77,78]. Eye drops containing 1% N-acetylcarnosine are marketed by Innovative Vision Products, but they are not currently approved by the FDA for treating cataracts.

#### 4.1.5. Quercetin

Quercetin is a flavonoid with scavenging and chelating properties. The scavenging action of quercetin may be attributed to the phenolic hydroxyl group in its structure. This group can neutralize free radicals by donating active hydrogen, transforming them into highly stable radicals, which helps prevent lipid peroxidation. Additionally, quercetin’s antioxidant activity can also be linked to its interaction with various enzymes [150]. Its chelating properties allow it to inhibit proteolytic activity and prevent excess calcium ions [81], protecting against opacification induced by calcium content dysregulation.

The ability of quercetin to protect the lens from oxidative damage and prevent cataract formation has been determined in various in vitro studies and animal models [82,83,84,85,86]. Quercetin application ameliorated the opacity induced by selenite treatment in an ex vivo rat lens model [84], as well as in an in vivo rat selenite cataract model [82,83].

Besides its chelating actions and antioxidant properties, quercetin can hinder aldose reductase and non-enzymatic glycation, playing an essential role in diabetic cataract treatment [87,88]. The inhibitory effect of quercetin on aldose reductase is related to its structural characteristics. Molecular docking studies demonstrated hydrogen bond formation between polar residues of quercetin and the aldose reductase amino acids Leu-300 and Thr-113, which are critical players in aldose reductase catalysis and inhibition [88].

One important drawback of quercetin use is its low chemical stability in aqueous solutions, showing rapid metabolism to 3′-O-methylquercetin by catechol-O-methyl transferase in the lens. However, despite its metabolism, it seems to retain its protective action [151]. Another main limitation of quercetin is its low bioavailability. To improve bioavailability and therapeutic actions, chitosan nanoparticle delivery systems have been designated and evaluated in cataract animal models [89]. The use of chitosan nanoparticles did not induce irritation/ocular toxicity and significantly increased corneal penetration and ocular distribution of quercetin; however, the anti-cataract effect was only modest [89].

#### 4.1.6. Chlorogenic Acid

Chlorogenic acid is a polyphenol formed by the condensation of quinic acid and caffeic acid that displays interesting antioxidant and anti-inflammatory biological activities [152] but exhibits a low oral bioavailability.

Chlorogenic acid significantly reduced ROS levels and inhibited apoptosis by decreasing the BAX/BCL-2 ratio in human lens epithelial cells treated with H_2_O_2_ [90]. Moreover, in rabbit lenses ex vivo, treatment with chlorogenic acid prevented lens opacity induced by hydrogen peroxide in a dose-dependent manner [90]. Chlorogenic acid is present in high concentrations in blueberry leaf extract, which showed a protective effect from oxidative damage triggered by selenite rat lenses [91]. Particularly, increased activity of antioxidant enzymes and GSH levels were detected with the use of the extract, as well as the maintenance of calpain activity at physiological levels preserving lens crystallin integrity [91]. However, since the blueberry leaf extract contains other polyphenols (quercetin, isoquercetin, hyperoside, and rutin), the beneficial effect on cataractogenesis progression cannot be exclusively attributed to chlorogenic acid.

On the other hand, chlorogenic acid can reduce diabetes-induced lens opacity by blocking aldose reductase and decreasing sorbitol accumulation in diabetic lenses [92,93]. Collectively, these findings suggest the potential of chlorogenic acid in cataract prevention and treatment, although additional research efforts to improve its bioavailability and clinical trials are necessary for future ophthalmic applications.

#### 4.1.7. Curcumin

The protective role of curcumin against oxidative stress, which delays cataract formation, has been evaluated in several cataract models. In selenium-induced oxidative stress cataract models, curcumin was able to abolish the reduction in enzymatic and non-enzymatic antioxidants [94]. Particularly, curcumin ameliorated the decrease in catalase, superoxide dismutase, and glutathione peroxidase in rats treated with sodium selenite [95,96,97]. Regarding non-enzymatic antioxidants, curcumin treatment prevented the alteration in reduced glutathione levels and vitamins C and E induced by sodium selenite [98]. A similar effect preserving the levels of reduced glutathione and vitamin C was demonstrated in rats with diabetic cataracts caused by streptozotocin [97,99]. Likewise, curcumin counteracted lipid peroxidation in both experimental cataract models [95,100].

Additionally, the contribution of curcumin in posterior capsule opacification attenuation has been suggested, considering its ability to inhibit proliferation and epithelial–mesenchymal transition by different pathways [101,102]. Thus, curcumin suppressed TGF-β-induced epithelial–mesenchymal transition through the blockade of the TGF-β/Smad pathway [102] and potassium voltage-gated channel subfamily Q member 1 opposite strand/antisense transcript 1/miR-377-3p/COL1A2 signaling regulation [101].

To circumvent the poor water solubility of curcumin and improve its bioavailability, new strategies based on nanotechnological approaches have been performed with varying success [58,103]. Curcumin was efficiently loaded into novel lipid–cyclodextrin-based nanoparticles, but in vitro assays revealed an inconsistent protective action of curcumin nanoparticles on H_2_O_2_-induced opacity in bovine lenses [58]. On the contrary, Grama et al. [103] reported that curcumin encapsulated in polymeric nanoparticles had higher oral bioavailability and a more pronounced effect in delaying cataract formation in diabetic rats compared to free curcumin [103].

#### 4.1.8. Disulfiram

Disulfiram is a dimer of diethyldithiocarbamate (a nitric oxide inhibitor) with antioxidant activity and anti-cataract potential in cataract rat models [104]. However, its poor water solubility of disulfiram can limit its use in ophthalmic delivery in the form of conventional eye drops, as it struggles to penetrate the cornea and achieve the required drug concentration. To overcome this limitation, several alternative ocular delivery systems have been developed. A formulation of disulfiram in 2-hydroxypropyl-β-cyclodextrin and methylcellulose produced a delay in cataract formation in a recessive-type hereditary strain of cataractous rats by attenuating the increase in the nitric oxide and lipid peroxidation levels [105]. Disulfiram nanoparticles also exhibited superior penetration capacity at the ocular level and residence time in the lens compared to free disulfiram, although its ophthalmic benefits on cataract prevention were not examined [106]. The efficiency of lipid emulsions for disulfiram delivery was also tested [107]. Disulfiram octo-arginine-modified lipid emulsions showed a high corneal penetration and a strong effect on delaying selenium-induced cataract generation [107].

#### 4.1.9. Metformin

Metformin is a drug used in type 2 diabetes treatment with anti-aging and anti-cataracts properties [120,121]. In fact, the prevalence of cataracts in diabetic patients treated with metformin seems to be lower than that found in non-users [122].

Metformin reduced oxidative stress-induced senescence of human lens epithelial cells by AMPK (5′ AMP-activated protein kinase) activation and autophagy activation [123,124]. Furthermore, autophagic flux restoration induced by metformin can be also related to sirtuin type 1 activation [125]. Similarly, in an aged mouse model, metformin alleviated the senescence of lens epithelial cells and inhibited the cloudiness of the lens by activating AMPK and enhancing autophagy [120].

Metformin also plays a defensive role in posterior capsule opacification [121]. Posterior capsule opacification results in fibrotic cataract appearance and is considered the major postoperative secondary loss of vision after cataract surgery [153]. A key pathological event in fibrotic cataract appearance is epithelial to mesenchymal transition. Metformin was able to inhibit the TGF-β2-Smad2/3 pathway, preventing epithelial to mesenchymal transition [121]. This notion about the preventive effect of metformin on posterior capsule opacification was also supported by the significantly lower risk for posterior capsule opacification development and subsequent Nd:YAG laser capsulotomy found in diabetic patients taking metformin [126].

#### 4.1.10. Vitamins C, D, and E

Vitamin C (ascorbic acid), a water-soluble antioxidant, is present in high concentrations in the aqueous humor and within the lens itself, where it functions to neutralize ROS and regenerate other antioxidants such as vitamin E and GSH. Experimental studies in animal models have demonstrated that vitamin C supplementation can attenuate oxidative damage and delay the progression of cataractogenesis by preserving lens transparency and preventing protein aggregation [108,109]. Vitamin C is not produced in humans and it must be obtained from the diet. Some epidemiological studies suggested that higher dietary intake or plasma concentrations of vitamin C exhibit a reduced risk of age-related cataract development, particularly nuclear and cortical subtypes [110,111]. Nevertheless, clinical trials examining the efficacy of vitamin C supplementation have produced variable outcomes [154], with some studies reporting modest protective effects [139] while others fail to demonstrate significant benefits [140]. Moreover, high doses of vitamin C (around 1000 mg/day) increased cataract risk in a Swedish cohort of women by 38% [141]. These discrepancies may stem from differences in study design, dosage, duration of supplementation, and baseline nutritional status of participants.

Vitamin D has emerged as a potential modulator in the prevention and treatment of cataracts, primarily due to its anti-inflammatory, antioxidant, and immunomodulatory properties. The active form of vitamin D, calcitriol (1,25-dihydroxyvitamin D_3_), exerts its effects through the vitamin D receptor, which is expressed in various ocular tissues, including the lens epithelium. Several epidemiological studies have reported an association between vitamin D deficiency and an increased risk of cataract development across diverse populations [112,113,114]. However, one study did not observe a significant relationship between serum vitamin D levels and the presence of nuclear cataracts [155]. The impact of dietary vitamin D supplementation on cataract progression has been examined in several studies. The Beaver Dam Eye Study reported a protective association between vitamin D intake and the development of nuclear cataracts [142]. In contrast, a recent randomized controlled trial involving 19,925 participants over a five-year period, in which individuals received 60,000 IU of vitamin D once per month, found no significant difference in cataract surgery rates between the supplementation group and the placebo group [143]. Consequently, current evidence regarding the role of vitamin D in cataract prevention remains inconclusive.

Vitamin E, a lipid-soluble antioxidant, inhibits lipid peroxidation and scavenges free radicals [156], thereby stabilizing lens fiber cell membranes and preserving lens clarity. Experimental studies in cataract animal models have demonstrated that vitamin E supplementation can delay cataract formation by mitigating oxidative damage and maintaining lens epithelial cell function (see [115] for review). Case–control studies [116,117] and cross-sectional studies [118,119] have reported an inverse association between dietary or serum levels of vitamin E and the risk of age-related cataract development. However, evidence from prospective cohort studies and randomized controlled trials has been inconsistent. While some studies have observed a protective effect of vitamin E [145], others have found no significant benefit [140,144,146]. Variability in study populations, vitamin E dosage, formulation, and duration of supplementation may account for these discrepancies.

Overall, while vitamins C, D, and E show promise as part of a multifaceted approach to cataract prevention, further well-designed, large-scale randomized controlled trials are necessary to clarify their clinical utility and establish standardized guidelines for their use in cataract prevention.

### 4.2. Aldose Reductase Inhibitors

Given the relevance of aldose reductase action in the development of diabetic cataracts, the use of aldose reductase inhibitors has been suggested as a potential effective drug treatment against cataracts (Table 1).

Promising aldose reductase inhibitors (ARIs) (sorbinil, tolrestat, and zopolrestat) showed valuable results in animal models [157]; however, they were discontinued during clinical trials due to limited efficacy and significant adverse effects [158]. More recently, oral treatment with the aldose reductase inhibitor diosgenin decreased aldose reductase activity in galactosemic rats, and galactitol concentration delayed lens opacification in the animals [127]. Additionally, Kinostat, another promising aldose reductase inhibitor, demonstrated potent efficacy against cataract formation in diabetic dogs and was anticipated to obtain FDA approval by 2017 [128]. Although these inhibitors revealed significant efficacy in mitigating diabetic cataracts in animal models, their effectiveness as therapeutic interventions for diabetic cataracts in human patients is limited [128]. The disparity of ARIs in alleviating cataract progression between animal models and humans can be related to interspecies variations in aldose reductase activity and distinct mechanisms involved in glucose metabolism.

### 4.3. Chaperon Peptides

A fundamental event in cataract formation is the loss of solubility of crystallin proteins, which are the most abundant proteins in the lens, resulting in aggregate formation. Within crystallins, α-crystallin, in addition to its structural role, has chaperone activity by inhibiting the aggregation of other proteins partially unfolded by oxidation and other stressful factors [159]. Considering the importance of α-crystallin (formed by two subunits, A and B) for maintaining the structural organization of the lens, synthetic peptides derived from this protein (mini-αA and mini-αB) have been generated, proving to be equally effective in preventing protein aggregation and precipitation [129] (Table 1). Moreover, the mini-αA peptide chaperone also appears to have antioxidant properties, capable of preventing copper-mediated ascorbic acid oxidation [130]. Additionally, it has been suggested that mini-αA and mini-αB chaperones may act as anti-apoptotic agents in in vitro studies [131]. Their therapeutic value in the treatment of cataracts has been demonstrated in a selenite-induced cataract model, with treated animal lenses showing inhibition of oxidative stress, lower protein insolubilization, and apoptosis reduction [160].

### 4.4. Protein Aggregation Inhibitors

#### 4.4.1. Oxysterols

Lanosterol and 25-hydroxycholesterol have been considered potential pharmacological agents capable of reversing protein aggregation in cataractous lenses. Lanosterol is an amphipathic compound and a cholesterol biosynthesis intermediate. Previous in vitro experiments showed that lanosterol was able to reduce protein aggregates [132]. Recent studies have demonstrated that lanosterol strongly binds to the C-terminal hydrophobic regions of γ-crystallin, preventing potential dimerization and aggregation of misfolded crystallins [133]. Lanosterol’s anti-aggregating capacity and its ability to partially restore lens transparency have been observed in ex vivo experiments with rabbit cataract lenses and in an animal model of senile cataracts in dogs [132]. Furthermore, congenital cataracts were linked to mutations in the lanosterol synthase gene, implicating endogenous lanosterol in maintaining lens homeostasis [161].

Another steroid derivative, 25-hydroxycholesterol, also possesses the ability to inhibit protein aggregation. In experiments using material obtained from phacoemulsification, after treatment with 25-hydroxycholesterol for 14 days, α-crystallins released from protein aggregates in greater quantities than lanosterol treatment was observed, although the latter also released β and γ-crystallins [134]. In murine models with hereditary and spontaneous cataracts, 25-hydroxycholesterol partially restored the solubility of α -crystallins and allowed the reversal of lens opacity [135].

However, Daszynski et al. [162] reported that neither lanosterol nor 25-hydroxycholesterol was able to increase soluble protein content or reduce aggregate load in ex vivo or in vitro cataract models, including human lenses. Particularly, lanosterol failed to reverse cataract formation in rat lenses subjected to physical trauma, osmotic stress, or Na^+^/K^+^ ATPase inhibition. Furthermore, the incubation of human lens fragments and homogenates with these oxysterols did not increase soluble protein levels or reduce insoluble protein aggregates. This lack of efficacy was also corroborated by docking studies where these oxysterols exhibited poor binding affinity to αB-crystallin and pharmacologically ineffective concentrations [162]. Shanmugam et al. [163] also failed to observe any effect of lanosterol on lens opacification reversal. The dense, cholesterol-rich membranes of lens fiber cells may further limit oxysterol penetration and bioactivity, highlighting inconsistencies in the efficacy of oxysterol-based therapy.

Complementing these biochemical and pharmacological studies, a recent genome-wide association study (GWAS) investigated the genetic relationship between lanosterol and cataract risk [164]. The results revealed no statistically significant associations between single nucleotide polymorphisms in the lanosterol synthase gene and cataract risk. These findings suggest that genetic variations influencing lanosterol synthesis do not correlate with cataract susceptibility, challenging the hypothesis that lanosterol plays a protective role in lens transparency maintenance.

#### 4.4.2. Rosmarinic Acid

This natural polyphenol found in various plants delays cataract formation and reduces the severity of lens opacification in a rat model with selenite-induced cataracts [136]. Along with its protein aggregation-preventing ability, rosmarinic acid has also been attributed to antioxidant and anti-inflammatory capacities, contributing to its beneficial effect in cataract treatment [137].

## 5. Conclusions

Cataracts remain a leading cause of reversible blindness globally, which is particularly exacerbated by aging and metabolic disorders like diabetes. While surgical intervention is the cornerstone of cataract treatment, its inherent complications, economic burden, and limitations in pediatric cases underscore the critical need for pharmacological alternatives that can delay onset, mitigate cataract progression, or, even, directly treat the cloudy lens.

Pharmacological treatments have been designed considering the pathological molecular mechanisms responsible for cataracts. These treatments include the use of antioxidants (e.g., resveratrol, curcumin, and quercetin), aldose reductase inhibitors, and protein aggregation inhibitors that have demonstrated potential utility in in vitro and in vivo studies. However, challenges related to drug stability, corneal penetration, and interspecies variations in therapeutic efficacy persist. The expansion of novel delivery systems, such as nanoparticles and lipid emulsions, has enhanced the ocular bioavailability and stability of some of these compounds. Thus, future innovations in nanotechnology-based delivery systems are required to overcome these barriers, enhancing drug stability, and improving therapeutic outcomes.

While preclinical studies have shown encouraging results, clinical validation of these treatments remains limited. Consequently, future research should also prioritize rigorous clinical trials to validate these pharmacological approaches, emphasizing patient stratification and cataract subtype-specific efficacy.

Additionally, continued exploration of molecular mechanisms, especially in the context of oxidative stress and protein aggregation, will be crucial in identifying new therapeutic targets. By addressing these challenges, pharmacological treatments could provide non-surgical therapeutic options to delay and/or treat cataracts, alleviating the socio-economic burden of cataracts and improving the quality of life for affected individuals worldwide.

## Figures and Tables

**Table 1:** Summary of drugs described in the manuscript and the delivery systems investigated for their ocular administration. References related to innovative drug delivery systems are shown in the right column.

Drug Name	Mechanism of Action	Refs.	Innovative Delivery System	Refs.
Resveratrol	Antioxidant activity Autophagy promotion Epithelial–mesenchymal transition prevention	[47,48,49,50,51,52,53,54,55,56]	Gold nanoparticles Lipid–cyclodextrin-based nanoparticles Chitosan nanoparticles	[57,58,59]
N-acetylcysteine, NACA, and diNACA	Antioxidant activity Apoptosis reduction	[60,61,62,63,64]	_	_
(*) Pirenoxine	Antioxidant activity	[65,66,67,68,69,70,71]	_	_
L-carnosine and N-acetylcarnosine (*)	Antioxidant activity Glycation inhibition Calpain-mediated proteolysis blockade	[72,73,74,75,76,77,78]	Lipid-based system with hyaluronic acid Gold nanoparticles	[79,80]
Quercetin	Antioxidant activity, chelating action preventing proteolytic activity, and inhibition of aldose reductase activity	[81,82,83,84,85,86,87,88]	Chitosan nanoparticles	[89]
Chlorogenic acid	Antioxidant activity, inhibition of apoptosis, and aldose reductase activity	[90,91,92,93]	_	_
Curcumin	Antioxidant activity, epithelial–mesenchymal transition inhibition	[58,94,95,96,97,98,99,100,101,102,103]	Lipid–cyclodextrin-based nanoparticles Polymeric nanoparticles	[58,103]
Disulfiram	Antioxidant activity	[104]	Formulation with 2-hydroxypropyl-β-cyclodextrin and methylcellulose Nanoparticles Octo-arginine-modified lipid emulsions	[105,106,107]
Vitamins C, D, and E	Antioxidant activity	[108,109,110,111,112,113,114,115,116,117,118,119]	_	_
Metformin	Autophagy flux restoration and epithelial–mesenchymal transition inhibition	[120,121,122,123,124,125,126]	_	_
Diosgenin	Aldose reductase inhibition	[127]	_	_
Kinostat	Aldose reductase inhibition	[128]	_	_
Chaperone peptides	Protein aggregation inhibition Antioxidant activity Anti-apoptotic action	[129,130,131]	_	_
Lanosterol	Protein aggregation inhibition	[132,133]	_	_
25-hydroxycholesterol	Protein aggregation inhibition	[134,135]	_	_
Rosmarinic acid	Protein aggregation inhibition Antioxidant activity	[136,137]	_	_

Drugs highlighted with an asterisk are in the market.

**Table 2:** Summary of clinical trials conducted for drugs described in the manuscript.

Drug Name	Year	Individuals Enrolled	Main Findings	Refs.
N-acetylcysteine	2021	30	The aim of this study was to evaluate the effect of Chitosan-N-Acetylcysteine on calculated IOL power prior to cataract surgery compared to preservative-free, hyaluronic-acid-containing eye drops, but no results were posted on ClinicalTrials.gov.	ClinicalTrials.gov ID NCT05049629
Pirenoxine	1983	14	No effect on the progress of cataracts.	[71]
	2004	72	Inhibition of lens opacification and cataract progression, especially in a group of patients up to 59 years of age.	[69]
N-acetylcarnosine	2001	49	Topographic studies demonstrated less density and corresponding areas of opacification in posterior subcapsular and cortical morphological regions and visual improvement in 87% of treated eyes compared to baseline.	[77]
	2009	75	Improvement in disability glare accompanied by independent improvement in acuity.	[76]
	2009	50,500	Efficacy in the nonsurgical treatment of age-related cataracts.	[138]
Vitamin C	2007	35,186	A higher vitamin C intake was associated with a reduced incidence of cataracts in both sexes.	[139]
	2010	11,545	Long-term (8 years) daily use of 500 mg of vitamin C had no notable beneficial effect on the risk of cataracts.	[140]
	2010	24,593 (all women)	Vitamin C supplements may be associated with a higher risk of age-related cataracts.	[141]
Vitamin D	1995	4926	Vitamin D intake was associated with protective action against cataract development.	[142]
	2023	19,925	Routinely supplementing older adults with high-dose vitamin D (60,000 IU once per month for a maximum of 5 years) is unlikely to reduce the need for cataract surgery.	[143]
Vitamin E	2004	1193	Vitamin E given for 4 years at a dose of 500 IU daily did not reduce the incidence or progression of cataracts.	[144]
	2005	408	Long-term use (5 years) of vitamin E supplements reduces the progression of age-related lens opacification.	[145]
	2010	11,545	Long-term (8 years) daily use of vitamin E (400 IU) had no notable beneficial effect on the risk of cataracts.	[140]
	2015	11,267	Long-term (5.6 years) daily supplementation with vitamin E (400 IU) is unlikely to have a large beneficial effect on age-related cataracts.	[146]

## Abbreviations

The following abbreviations are used in this manuscript:

UV	Ultraviolet
ROS	Reactive oxygen species
TRP	Tryptophan
GSH	Reduced glutathione
NRF2	Nuclear factor erythroid 2-related factor
BAX	Bcl-2-associated X protein
BCL-2	B-cell lymphoma-2
AR	Aldose reductase
SDH	Sorbitol dehydrogenase
NADH	Nicotinamide adenine dinucleotide
GSSG	Oxidized glutathione
AGEs	Advanced glycation end products
TGF-β	Transforming growth factor-β
α-SMA	smooth muscle actin
AMPK	5′ AMP-activated protein kinase
FDA	United States Food and Drug Administration
